# Computational and experimental analysis for the mismatched DNA recognition mechanism of *Taq*MutS

**DOI:** 10.1016/j.synbio.2026.10.011

**Published:** 2026-11-04

**Authors:** Wenyan Jiang, Yiyang Ding, Haojie Sun, Yuxuan Li, Xiao Liang, Quanshun Li

**Affiliations:** Key Laboratory for Molecular Enzymology and Engineering of Ministry of Education, School of Life Sciences, Jilin University, Changchun, 130012, China

**Keywords:** DNA synthesis, Error correction, *Taq*MutS, Mismatched DNA recognition, Molecular dynamics simulations

## Abstract

DNA holds great potential as a next-generation information storage material due to its high density, stability and low energy consumption. However, high synthesis errors severely limit its practical implementation. Enzymatic error correction based on *Thermus aquaticus* MutS (*Taq*MutS) is efficient and straightforward, whereas its severe non-specific binding to perfectly matched DNA drastically decreases the correction performance. In this work, we systematically analyzed the mechanism of mismatched DNA recognition by *Taq*MutS through alanine scanning mutagenesis, homologous substitution mutations and molecular dynamics simulations. The results revealed that *Taq*MutS recognized mismatched sites by sensing the local conformational strain of DNA. Specifically, a cooperative push-pull mechanism mediated by residues S57 and D472 modulated the π-stacking interactions between the key recognition residue F39 and DNA. Meanwhile, the electrostatic synergistic effects of residues K61 and R473 effectively regulated the non-specific DNA binding of *Taq*MutS. Based on these findings, two mutants (V57S and K61H) were constructed, which exhibited enhanced binding efficiency for mismatched DNA and reduced affinity for perfectly matched DNA. Collectively, this study provided useful error-correction tools for high-efficiency DNA synthesis and laid an important theoretical foundation for the rational design of mismatched DNA recognition proteins.

## Graphical abstract

**Figure:**
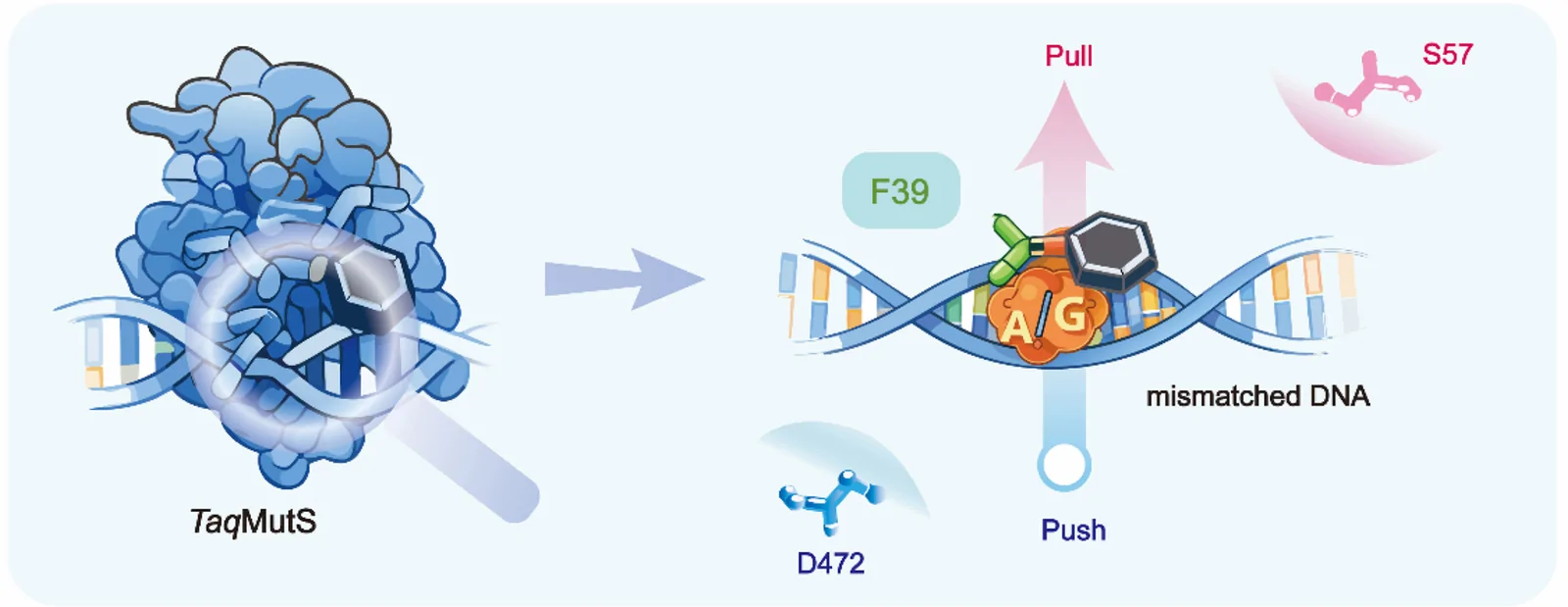

## 1 Introduction

DNA represents a promising next-generation information storage medium that transcends the physical limits of conventional storage technologies, owing to its ultrahigh storage density, favorable chemical stability and low energy consumption for storage [1], [2], [3]. However, key issues in the data writing process of DNA information storage, including high cost, low speed, and high error rate of DNA synthesis, still restrict the practical application of DNA as a data storage medium [4]. Among these issues, side reactions during DNA synthesis (*e.g.*, incomplete coupling and incorrect incorporation) result in an error rate as high as 1-3 errors per kilobase (kb), leading to reduced synthesis efficiency and increased production cost [5], [6]. Further, as errors accumulate exponentially with the DNA fragment length, the fraction of correct sequences within long oligonucleotide pools is often low, with only 5-60% of sequences being completely correct [7], [8]. Therefore, the adoption of error-correction technologies to reduce the synthesis error ratio can effectively improve the synthesis quality and lower the production cost [9]. Currently, molecular weight-based screening methods such as high-performance liquid chromatography and polyacrylamide gel electrophoresis, have been used to eliminate the length-mismatched erroneous sequences. However, these approaches are labor-intensive and ineffective against minor errors such as single-base deletions, insertions or substitutions [10], [11]. In addition, the hybridization selection technology can filter a large number of oligonucleotides, but the required number of oligonucleotides is actually doubled, resulting in high error-correction cost [12], [13]. In contrast, the mismatched DNA binding protein MutS enables the error correction and the isolation of diverse error types from synthetic DNA libraries, thereby exhibiting great potential in high-fidelity DNA synthesis [14].

MutS is an important component of the DNA mismatch repair (MMR) system in organisms, and it can recognize and bind diverse types of mismatched DNA substrates [15], [16]. Currently, there are two major types of MutS proteins used for error correction, including *Taq*MutS from *Thermus aquaticus* and *Eco*MutS from *Escherichia coli* [17], [18], [19], [20]. Among them, *Eco*MutS exhibits superior specific mismatched DNA recognition ability compared with *Taq*MutS, especially for the highest affinity for G-T mismatch [19]. However, *Eco*MutS has poor thermal stability, and its error-correction ability gradually decreases after 7 days, with the error-correction efficiency to less than 90% of original activity after 21 days [20]. In contrast, *Taq*MutS has higher stability and can maintain its original activity over a wide range of pH and temperature (0–70 °C), rendering it broadly applicable for error correction in DNA synthesis as well as the detection of genetic diseases [21], [22], [23], [24], [25]. Nevertheless, *Taq*MutS displays prominent non-specific binding during the DNA error-correction workflow, resulting in the misidentification and undesired removal of perfectly matched DNA as erroneous mismatched sequences, which significantly reduces the error-correction efficiency [9]. Accordingly, reducing non-specific recognition and enhancing specific binding of *Taq*MutS could improve the accuracy of error correction.

To improve the error-correction performance of *Taq*MutS, a comprehensive understanding of its structural features is critical to establish the molecular basis of mismatched DNA recognition. *Taq*MutS is composed of a homodimer, with each monomer containing five domains (I-V), and DNA molecule binds in the closed cavity between two subunits, mainly interacting with domains I and IV [26], [27]. Among these domains, F39 and E41 in domain I have been identified to play key roles in *in vitro* mismatch recognition, as they can form specific cross-links with heteroduplex DNA containing mismatched bases [28], [29], [30]. Similar to other MutS family proteins, *Taq*MutS undergoes a conformational change upon recognizing and binding to mismatched DNA, thereby forming a sliding clamp structure and interacting with other mismatch repair proteins to facilitate the DNA repair [31], [32], [33], [34]. However, the detailed mechanism underlying mismatched DNA recognition by *Taq*MutS has not been fully elucidated, especially the structural basis of its high non-specific binding.

In this study, the molecular basis of mismatched DNA recognition by *Taq*MutS was comprehensively studied. Sequence alignment and molecular dynamics (MD) simulations were utilized to screen critical recognition domains of *Taq*MutS, and electrophoretic mobility shift assays were conducted to analyze its non-specific binding profiles. Based on these findings, alanine scanning mutagenesis was carried out to identify decisive residues mediating the non-specific interaction, while homologous substitution was adopted to elucidate the molecular basis of its mismatched DNA detection.

## 2 Materials and methods

### 2.1 Materials

Tryptone, yeast extract, *E. coli* DH5α and BL21(DE3), single-stranded DNA (ssDNA) for the construction of mismatched DNA, and primers for site-directed mutagenesis were purchased from Sangon Biotech (Shanghai, China). Codon optimization and gene synthesis were conducted by GenScript (Nanjing, China). DNA polymerase was obtained from SparkJade (Shandong, China). Restriction endonuclease *Dpn*I was purchased from ThermoFisher (Waltham, MA, USA). Isopropyl β-D-1-thiogalactopyranoside (IPTG) was purchased from Sigma-Aldrich (Shanghai, China). 10× DNA/RNA Native Sample Loading Buffer and His-tag agarose magnetic beads were obtained from Beyotime (Shanghai, China).

### 2.2 Construction of plasmids and strains

The DNA sequences of *Taq*MutS (GenBank accession No.: WP_053767724) and *Eco*MutS (GenBank accession No.: AAC75775) were synthesized by GenScript (Nanjing, China). These sequences were inserted into pET-32a(+) plasmid, and the resulting recombinant plasmids were transformed into *E. coli* BL21(DE3) and *E. coli* DH5α, respectively. After the transformation, the bacterial cells were screened on LB medium supplemented with ampicillin (50 μg/mL).

### 2.3 Site-directed mutagenesis

Mutants were constructed using a site-directed mutagenesis kit from Beyotime (Shanghai, China). The primers were designed to target the desired mutation sites, and one-step PCR was carried out with the *Taq*MutS-encoding plasmid as the template to generate the mutant plasmids. The primers are listed in Table S1. The main PCR procedure was set as follows: pre-denaturation at 98 °C for 4 min; followed by 35 cycles of denaturation at 98 °C for 15 s, annealing at 75 °C for 15 s, and extension at 72 °C for 5 min. After the PCR amplification, the products were digested with *Dpn*I at 37 °C for 1 h, and then transformed into the competent *E. coli* BL21(DE3) cells. Successful mutation was verified *via* DNA sequencing, after which the mutant proteins were expressed and purified.

### 2.4 Expression and purification of MutS protein

The *E. coli* cells harboring MutS gene were inoculated into 50 mL of TB medium supplemented with 100 μg/mL ampicillin, and cultured at 37 °C for 4 h (180 rpm) until OD_600_ value reached 0.6. Subsequently, the culture temperature was adjusted to 18 °C, and 1 mM IPTG was added to induce the protein expression, followed by the continuous cultivation for 18 h. Bacterial cells were harvested by centrifugation at 8000 × *g* at 4 °C for 10 min, and the pellet was resuspended in Tris-HCl buffer (20 mM, pH 7.5). Cells were lysed by sonication, followed by centrifugation (8000 × *g*, 10 min). MutS protein was subsequently purified using Ni-NTA agarose affinity chromatography. The purity of MutS protein was analyzed by sodium dodecyl sulfate-polyacrylamide gel electrophoresis (SDS-PAGE), and the protein concentration was determined by Bradford method. Sequence-based solubility profiles were predicted *via* the CamSol method. Residue-level solubility scores spanning the mutation site (residues 468–476) were extracted for comparative analysis. Protein aggregation propensity was assessed using AGGRESCAN, in which two output parameters were applied to compare the aggregation propensity of WT and D472A, Total Area (TA) and normalized a4v sequence sum per 100 residues (Na4vSS).

### 2.5 Preparation of mismatched DNA

R-strand DNAs containing different types of mismatches and correct F-strand DNAs were separately dissolved in TE buffer (10 mM Tris, 0.1 mM EDTA, pH 7.5) to prepare single-stranded DNA (ssDNA) solutions with a concentration of 10 μM. Subsequently, these two solutions were mixed at a volume ratio of 1:1, and the mixture was treated at 95 °C for 5 min in a PCR thermocycler, then cooled down to 25 °C at a rate of 5 °C per minute and held at 25 °C for 5 min. Double-stranded DNA (dsDNA) solutions containing mismatches were obtained through this gradual annealing process. The sequences of R-strand and F-strand DNAs are listed in Table S2.

### 2.6 Electrophoretic mobility shift assay

Briefly, a reaction mixture was prepared by combining 10 μL of MutS protein solution (0.5 mg/mL), 2 μL of mismatched DNA solution, 2 μL of binding buffer (0.1 M KCl, 1 mM MgCl_2_, 0.25 mM EDTA, 0.5 mM DTT, 5% v/v glycerol, 50 mM Tris-HCl, pH 8.5), and 8 μL of ultrapure water. The mixture was incubated at 37 °C for 30 min to ensure sufficient binding of MutS protein with dsDNA. Then 10× DNA/RNA Native Sample Loading Buffer was added to the reaction system as recommended by the manufacturer. The samples were loaded onto a 10% non-denaturing polyacrylamide gel (prepared with 0.5× TE buffer) and electrophoresed at a constant voltage of 100 V for 2 h at 4 °C. Thereafter, the gel was stained by soaking in Gelblue nucleic acid dye (5×) for 10 min, followed by the decolorization with deionized water. The images were captured using a Universal Hood II Gel Imaging System (Bio-Rad, USA), and the gray-scale analysis of images was performed using ImageJ 1.53 software. The grayscale value of DNA band without MutS protein was set as 100%, and the binding rate of MutS protein to different dsDNA samples was calculated. All experiments were performed in triplicate, and results are presented as mean ± standard deviation.

### 2.7 Molecular dynamics simulation

The three-dimensional structures of the systems consisting of MutS proteins (including their variants) and mismatched DNA were constructed using AlphaFold3. Gaussian accelerated molecular dynamics (GaMD) simulation for all the model systems was performed using the Graphics Processing Unit (GPU)-accelerated version of Amber 20. The ff14SB force field, OL15 force field, and General Amber Force Field (GAFF) were employed to generate the parameters and topological structures of proteins and DNA, respectively. A water box was added to each model using the TIP3P force field, with a minimum distance of 10 Å maintained between the enzyme surface and the edge of the water box. Sodium ions were added to ensure the electrical neutrality of the system. Prior to formal simulations, all the systems underwent energy minimization using the steepest descent method and conjugate gradient algorithm. After the minimization, each system was gradually heated from 0 K to 298 K *via* Langevin dynamics, followed by the equilibrium in the NVT ensemble (constant number of particles, volume, and temperature). Additionally, the CPPTRAJ module in Amber 20 and the VMD software were used for the analysis and visualization of all the MD trajectories.

### 2.8 Temperature-dependent binding and storage stability assays

For temperature-dependent binding analysis, *Taq*MutS and its variants were incubated with mismatched DNA across a temperature gradient of 20–70 °C for 30 min. The mismatched DNA binding rate for each protein was determined by EMSA using A/G mismatch-bearing DNA as the substrate. For the storage stability, protein samples were stored at 4 °C over a 24-day period, with aliquots harvested at Day 0, 3, 6, 12, 18 and 24. At each time point, the proteins were incubated with A/G mismatch-bearing DNA at 37 °C for 30 min, and their mismatched DNA binding rates were measured using EMSA.

### 2.9 Assessment of error-removal performance

Prior to the error-removal assay, varying volumes of His-tag magnetic agarose beads (0, 10, 20, 30, and 40 μL) were tested to optimize the capture of *Taq*MutS. Unbound proteins in the supernatant were quantified after the magnetic separation. The optimal bead volume in the pre-experiment was adopted in the subsequent error-removal procedure. A defined DNA pool was prepared with perfectly-matched DNA and four distinct mismatch-bearing DNA sequences to evaluate the error-removal performance. The sequences of perfectly-matched DNA and mismatched substrates are listed in Table S3. The molar ratio of perfectly-matched DNA to the four mismatch-containing substrates was set to 80:5:5:5:5. The DNA pool was incubated with WT or its variants for 30 min under the conditions described in section 2.6. The His-tag magnetic agarose beads were added to the mixture, followed by an incubation for 30 min to form the *Taq*MutS-mismatched DNA complexes. The samples were subjected to magnetic separation for 5 min, after which the supernatants were collected for next-generation sequencing analysis.

## 3 Results and discussion

### 3.1 Evaluation of mismatched DNA recognition ability of EcoMutS and TaqMutS

*Eco*MutS and *Taq*MutS were expressed in *E. coli* BL21(DE3) and purified through Ni-NTA agarose affinity chromatography, and the protein purity was determined by SDS-PAGE. As shown in Figs. S1 and S2, distinct and homogeneous protein bands at molecular weights of 98 kDa (*Eco*MutS) and 95 kDa (*Taq*MutS) could be observed after the elution with 150 mM imidazole, which were consistent with their theoretical molecular weights. These results demonstrated that *Eco*MutS and *Taq*MutS proteins have been successfully obtained with high purity, providing a solid foundation for subsequent studies. To systematically compare the mismatched DNA-recognition capabilities of *Eco*MutS and *Taq*MutS across diverse DNA error types, six representative replication lesions (A/G, C/T, G/A, G/T, T/C mismatches and single-base deletion) were selected, and their mismatch binding capacities were quantitatively analyzed by electrophoretic mobility shift assays. As illustrated in Fig. 1, the binding efficiencies of *Taq*MutS toward A/G, C/T, G/A, G/T, T/C mismatches and single-base deletion were measured to be 78.6%, 36.8%, 59.1%, 59.9%, 69.7% and 71.7%, respectively, which were 4.5-26.2% lower than the corresponding values of *Eco*MutS. It is well-established that *Eco*MutS exhibits strong binding affinity toward G/T mismatches, whereas this binding behavior is not observed for *Taq*MutS. This discrepancy can be attributed to two key factors. First, although *Eco*MutS and *Taq*MutS are homologous proteins, differences in their amino-acid sequences and structural flexibility will yield distinct substrate-binding preferences. Second, the sequence-context effects originating from the flanking DNA around mismatch sites may alter the duplex conformation and further modulate the protein-DNA binding affinity. Notably, the non-specific binding level of *Taq*MutS to fully matched DNA reached 39.6%, which was considerably higher than that of *Eco*MutS (7.2%). Compared with *Eco*MutS, *Taq*MutS exhibited lower mismatched DNA recognition efficiency and stronger non-specific binding ability, which was consistent with the previously reported results [9], [19]. Nevertheless, *Taq*MutS showed remarkable thermostability, showing irreplaceable advantages in biotechnological applications. Thus, *Taq*MutS was selected as the model protein to analyze its mismatched DNA recognition mechanism.

**Fig. 1:**
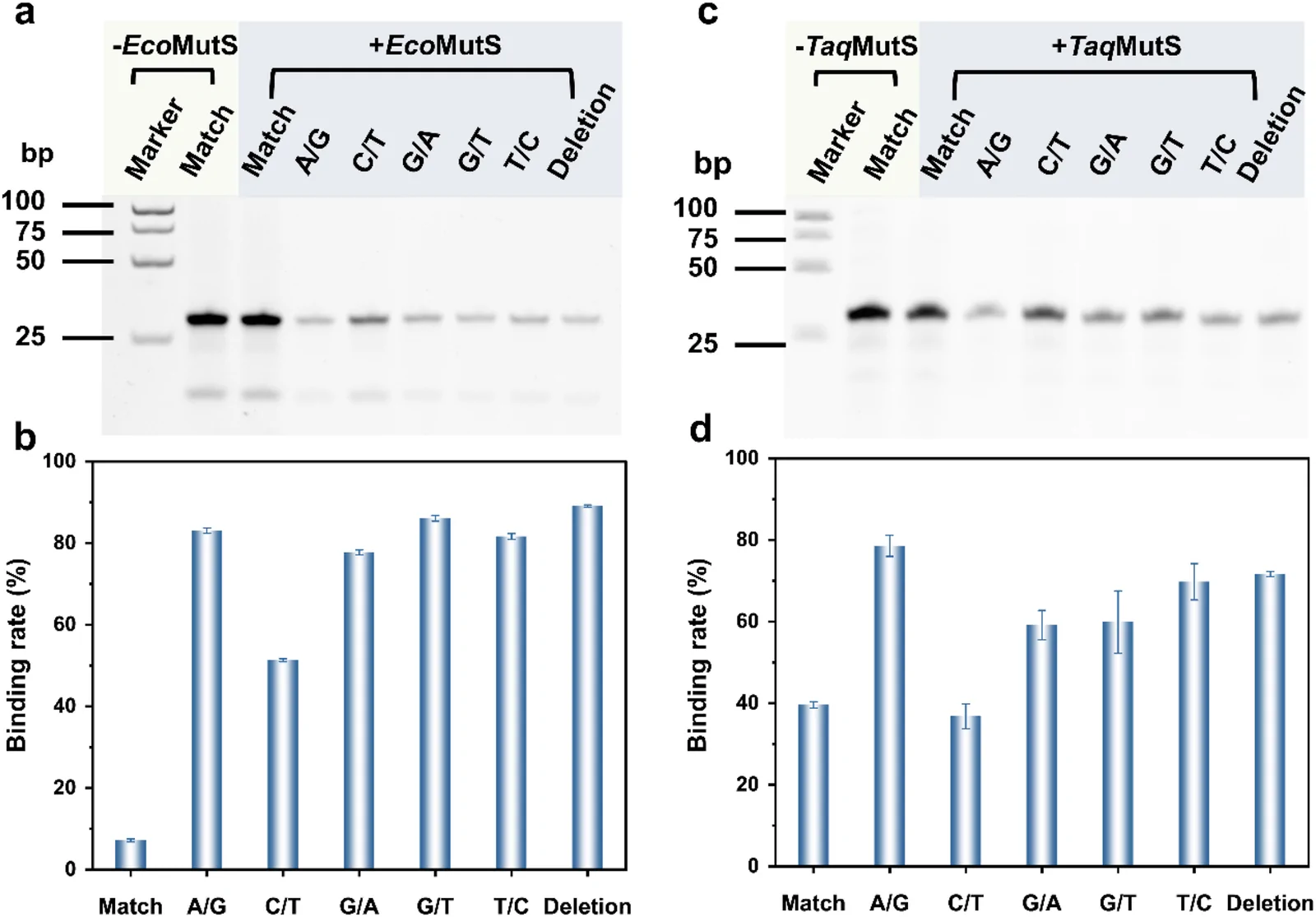
The mismatched DNA recognition ability of *Eco*MutS and *Taq*MutS. (a) Non-denaturing polyacrylamide gel results (b) and gray-scale quantitative results of *Eco*MutS binding to different mismatched DNAs. (c) Non-denaturing polyacrylamide gel results and (d) gray-scale quantitative results of *Taq*MutS binding to different mismatched DNAs.

### *3.2* *Identification of key recognition sites in TaqMutS using alanine scanning* mutagenesis

Since *Taq*MutS exhibited the highest binding affinity toward A/G mismatches, this mismatch type was selected for subsequent computational simulation. To identify key residues involved in mismatched DNA recognition, dynamic conformational properties and intermolecular interaction energy differences between *Taq*MutS bound to fully matched DNA and A/G mismatched DNA were analyzed. A triplet region consisting of the core mismatched base pair (DG9:DT22) and its adjacent upstream and downstream base pairs (DC8:DG23 and DC10:DG21) was chosen, and 23 residues within a 5 Å radius of this region were investigated (Fig. 2a). Subsequently, the MM/GBSA (molecular mechanics/generalized born surface area) residue decomposition method was applied to the *Taq*MutS-matched DNA and *Taq*MutS-mismatched DNA complex systems. Residues with |*ΔΔ*G| < 1 kcal/mol were excluded from further analysis (Fig. 2b). Combined with evolutionary conservation analysis results of MutS family (Fig. 2c), highly conserved functional sites (F39, Y40 and E41) were further eliminated. Accordingly, 6 potential residues affecting the mismatched DNA binding of *Taq*MutS were screened out, namely V36, V57, K61, R76, D472 and R473.

**Fig. 2:**
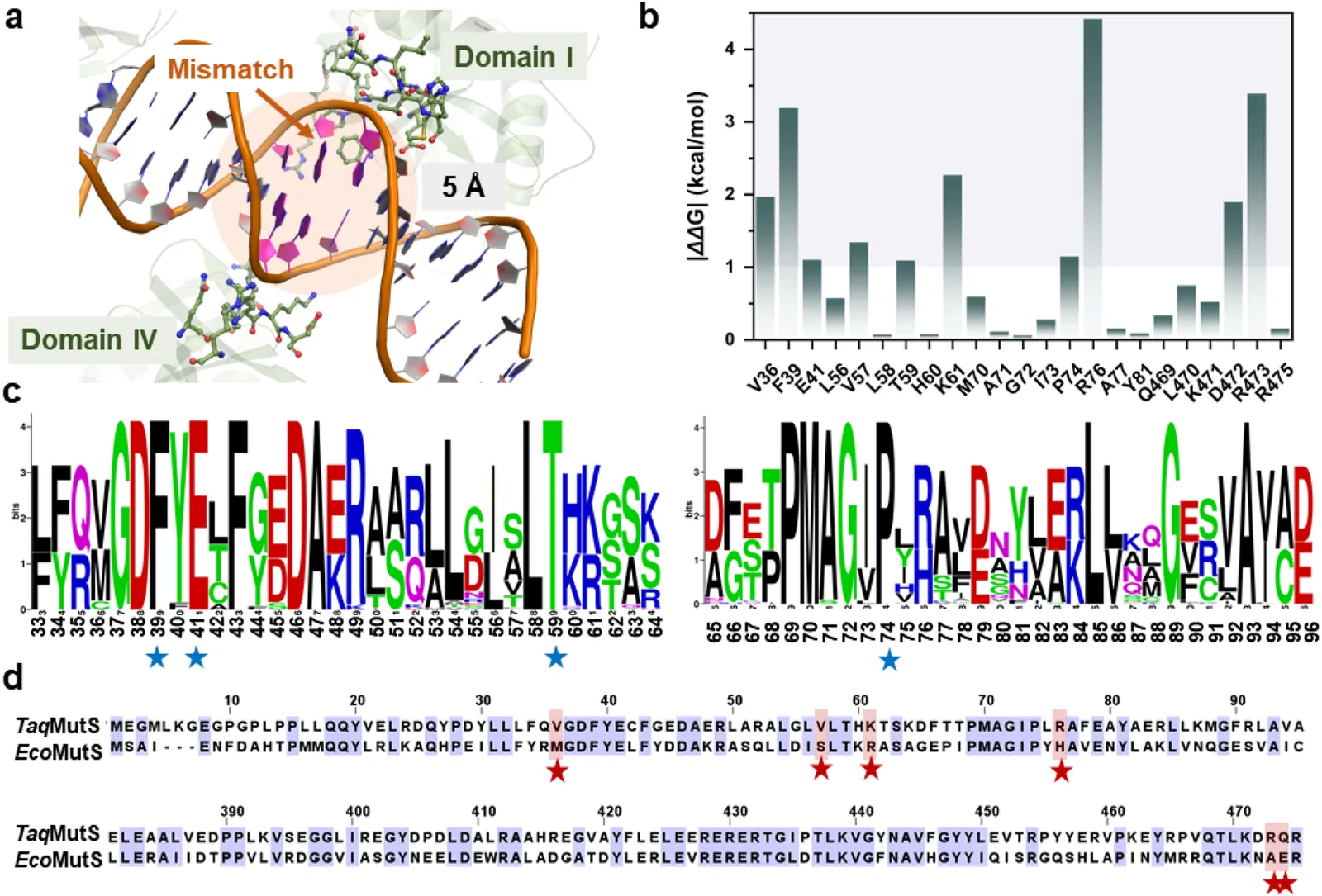
Alanine scanning mutagenesis and the design of homologous substitution mutation sites. (a) Structural visualization of amino acid residues within 5 Å of the mismatched base pairs and their adjacent base pairs. (b) Free energy decomposition contribution of WT binding to correct and A/G mismatched DNA using the MM/GBSA binding method, where |*ΔΔ*G| = |*Δ*G_correct - *Δ*G_A/G mismatch|. (c) Sequence conservation analysis of the MutS family. (d) Comparative sequence analysis of *Taq*MutS and *Eco*MutS homologs.

Alanine scanning mutagenesis was then performed to characterize the effects of these sites on the mismatched DNA recognition capacity of *Taq*MutS. The purification performance of these mutants was evaluated by SDS-PAGE and protein concentration measurements (Fig. S3). Most mutants could be successfully purified with the concentrations of 0.38–0.70 mg/mL, whereas D472A showed a markedly fainter protein band and a substantially lower protein concentration (0.07 mg/mL). Structural analysis showed that D472 formed a local hydrogen-bond interaction with R473 in *Taq*MutS, whereas the mutation D472A disrupts this interaction network (Fig. S4a and S4b). Further, residue-level solubility and aggregation propensity analyses were performed to quantitatively monitor these structural alterations. Compared to WT, the mutant D472A exhibited lower predicted solubility across the residues surrounding the 472 site (Fig. S4c). To quantify the mutation-driven increase in aggregation propensity, two well-established sequence-derived computational indicators, TA and Na4vSS, were utilized for comparative analysis. TA reflects the overall hydrophobic aggregation-prone area of local protein regions, while Na4vSS quantifies the residue-specific intrinsic aggregation propensity. This mutation elevated both aggregation-related parameters: the TA value increased from −23 to −21, and the Na4vSS value rose from −4.9 to −4.6 (Fig. S4d). Collectively, these computational analyses confirmed that the D472A mutation reduced the solubility of this local loop and increased its intrinsic aggregation propensity. The binding ratios of all mutants to fully matched DNA markedly decreased to < 20%, demonstrating that these residues were associated with the non-specific binding of *Taq*MutS (Fig. 3 and S5). In addition, all the mutants displayed obvious impairments in mismatched DNA binding ability, especially the mutants V36A and V57A (Fig. 3a and b). Specifically, the V36A mutant completely lost the binding capacity toward A/G and C/T mismatches (binding ratio < 10%), and its binding efficiencies toward other mismatches (G/T, T/C and single-base deletion) were also reduced to 40–65%. The V57A mutant only retained the binding activity toward T/C mismatches (65.0%) and single-base deletion (64.2%), while its binding toward A/G, C/T and G/T mismatches was almost completely abolished. Although the mutants K61A and R76A decreased the protein's binding affinity for the correctly base-paired DNA, they also weakened the binding capacity to the mismatched DNA substrates (Fig. 3c and d). Overall, these results demonstrated that V36, V57, K61, R76 and R473 were key residues regulating the mismatched DNA recognition of *Taq*MutS. Among them, V36 and V57 exerted particularly prominent effects on the mismatched DNA binding ability of *Taq*MutS. Nevertheless, the mutant D472A could not be obtained in the present work, leaving its exact functional contribution to mismatched DNA recognition uncharacterized.

**Fig. 3:**
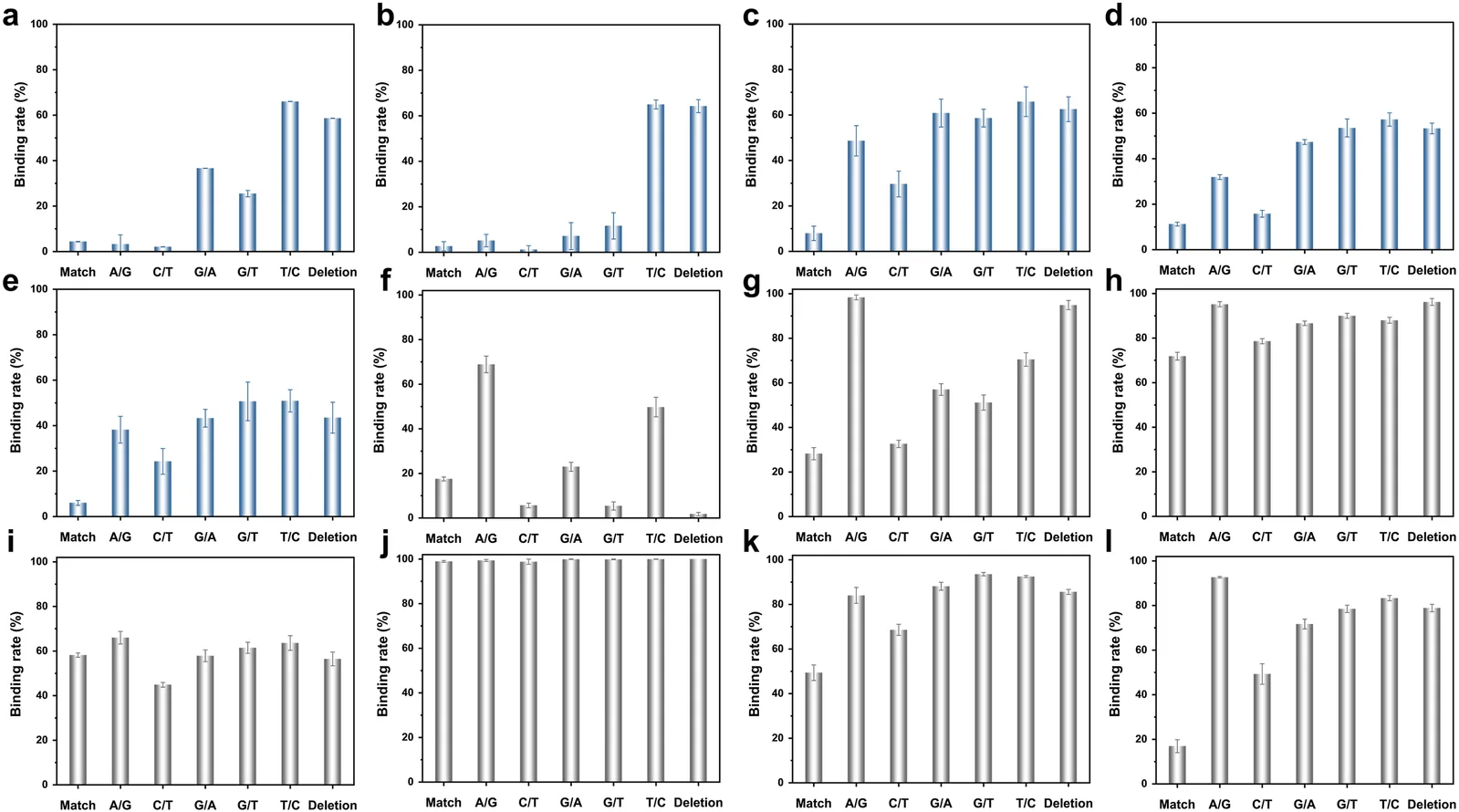
Gray-scale quantitative results of *Taq*MutS mutants binding to different mismatched DNAs: (a) V36A; (b) V57A; (c) K61A; (d) R76A; (e) R473A; (f) V36M; (g) V57S; (h) K61R; (i) R76H; (j) D472N; (k) D472E; (l) K61H.

### 3.3 Key sites for mismatched DNA recognition of TaqMutS by homologous substitutions

*Eco*MutS displayed superior performance over *Taq*MutS in the mismatched DNA recognition, featuring both enhanced binding affinities toward diverse mismatched DNA substrates and reduced non-specific binding to fully matched DNA. Accordingly, homologous substitution mutations (V36M, V57S, K61R, R76H, D472N and R473A) were designed at the identified key residues on the basis of sequence alignment between *Eco*MutS and *Taq*MutS (Fig. 2d). Similarly, successful purification of these mutants was confirmed through SDS-PAGE and protein concentration quantification (Fig. S3). Their binding efficiencies toward various DNA substrates were evaluated. Among them, the functional properties of R473A had been systematically characterized through alanine scanning mutagenesis. As presented in Fig. 3f–j, the binding ratio of V36M to fully matched DNA decreased to 17.5%, whereas its binding efficiencies toward C/T, G/T mismatches and single-base deletion DNA dropped sharply to below 10%. The non-specific binding efficiency of V57S declined significantly to 28.2%, while its recognition efficiencies toward A/G mismatch and single-base deletion DNA were greatly improved to 98.4% and 94.9%, respectively (Fig. 3g). Although K61R displayed high binding efficiencies toward all mismatch types (80.0–95.2%), its non-specific binding efficiency increased to 71.8% (Fig. 3h). Notably, D472N exhibited indiscriminate binding toward all DNA substrates with efficiencies up to 98%, indicating that it completely lost the ability to discriminate the matched and mismatched DNA (Fig. 3j). Based on these findings, Gaussian accelerated molecular dynamics (GaMD) simulations were subsequently conducted to analyze how the mutations at these key residues modulated the conformational dynamics of *Taq*MutS, thereby deciphering the molecular basis governing its mismatched DNA recognition behavior.

### 3.4 Local conformational strain affects the mismatched DNA recognition of TaqMutS

It has been reported that DNA mismatches could induce local distortion of the double helix centered on mismatched base pairs, and the mismatched DNA-recognition mechanism of *Taq*MutS is considered to rely on such structural distortions [35], [36]. Accordingly, taking the A/G mismatched base pair as the core, its adjacent upstream and downstream base pairs were selected. Hydrogen bond numbers and stacking angles among these base pairs were analyzed to explore the local conformational distortion of mismatched DNA bound to wild-type (WT) *Taq*MutS and its mutants. As shown in Figs. S6 and S7, 7–9 hydrogen bonds were formed among the three base pairs in WT-matched DNA complex. Meanwhile, the base stacking angles of DC8, DA9 and DC10 ranged from 150° to 180°, displaying a parallel stacking tendency, which demonstrated that these base pairs maintained a canonical double-helical structure. Conversely, the number of hydrogen bonds among these three base pairs decreased to 4–7 in the WT-A/G mismatched DNA complex (Fig. 4a and b). For the V57S mutant, the hydrogen bond number further declined to 3-6 (Fig. 4c and d), indicating notable local distortions at the mismatch site and weakened hydrogen-bonding interactions between mismatched bases. In addition, the π-stacking angles between the mismatched base DG9 and its flanking bases were mainly distributed at 125° and 150° in the WT-A/G mismatched DNA complex (Fig. 4e and f). In contrast, the corresponding π-stacking angles in V57S were predominantly at 120° (Fig. 4e and g), revealing the disruption of parallel base stacking and obvious local distortion of mismatched DNA. Collectively, these results demonstrated that the local bulging distortion within mismatched DNA serves as the key determinant for recognition by *Taq*MutS, and the distortion degree was closely correlated with its binding efficiency.

**Fig. 4:**
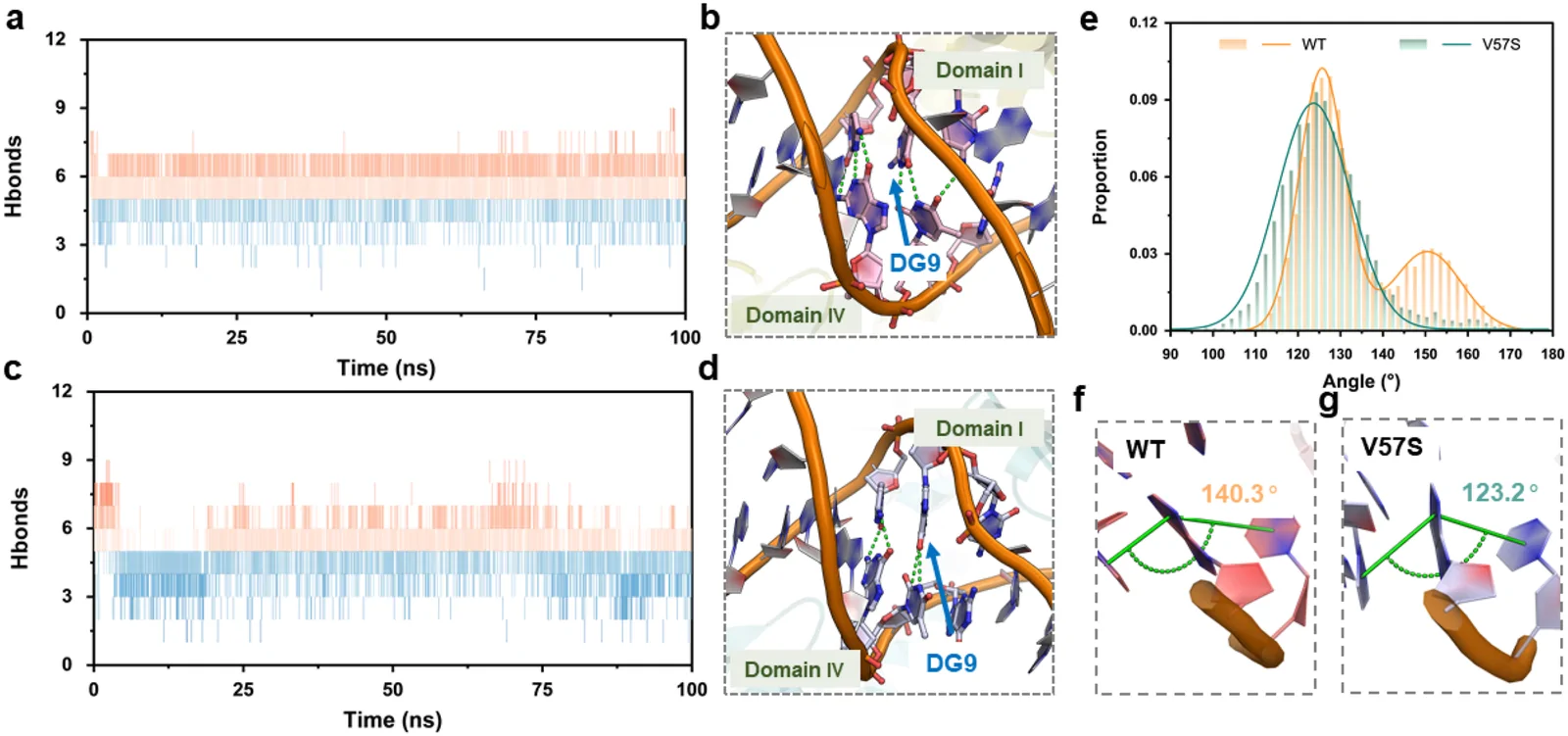
Local deformation analysis of mismatched DNA in the WT and V57S systems. The number of hydrogen bonds between DC8, DG9, and DC10 and their paired bases in WT and A/G mismatched DNA systems during 100-ns GaMD simulation (a) and the visualized structure (b); The number of hydrogen bonds between DC8, DG9, and DC10 and their paired bases in the V57S and A/G mismatched DNA systems during 100-ns GaMD simulation (c) and the visualized structure (d); The probability distribution of π-stacking angles between the DC8, DG9, and DC10 base loops (e) and the visualized structure of WT (f) and V57S (g).

### 3.5 Push-pull mechanism involving the cooperative regulation of F39-mediated mismatched DNA recognition by V57 and D472

In the WT-mismatched DNA complex, we found that the distance between V57@CG and DG9@P ranged from 5 to 6 Å (Fig. 5a and b). In contrast, the distance between S57@OG and DG9@P was distributed from 2 to 5 Å in the V57S-mismatched DNA complex, which was attributed to the hydrogen bond formed between the side-chain hydroxyl group of S57 and the phosphate group of the mismatched DNA backbone (Fig. 5a and c). Since V/S57 was spatially adjacent to F39, the hydrogen bond interaction between S57 and mismatched DNA could push the mismatched DNA closer to F39. As F39 has been considered as a key residue for mismatched DNA recognition, we further analyzed the interaction between F39 and mismatched DNA. In the V57S-mismatched DNA complex, the distance between the benzene ring of F39 and the base ring of DC8 was approximately 4 Å, while the distance between the benzene ring of F39 and the base ring of DG9 was maintained at around 6 Å (Fig. 5d–f and S8). In contrast, the distances between the benzene ring of F39 and the base rings of DC8 and DG9 were both maintained at approximately 7 Å in the WT-mismatched DNA complex (Fig. 5d and g). These results demonstrated that the π-stacking interaction between F39 and the DNA mismatch site was stronger in V57S, which was caused by the hydrogen bond between S57 and mismatched DNA to shorten the distance and thereby stabilize the binding of F39. Notably, the distance between the benzene ring of F39 and the base of DA9 was more than 10 Å in the WT-matched DNA complex (Fig. S9), further indicating that the specific recognition of mismatched DNA by *Taq*MutS was highly associated with the interaction between F39 and the mismatch site.

**Fig. 5:**
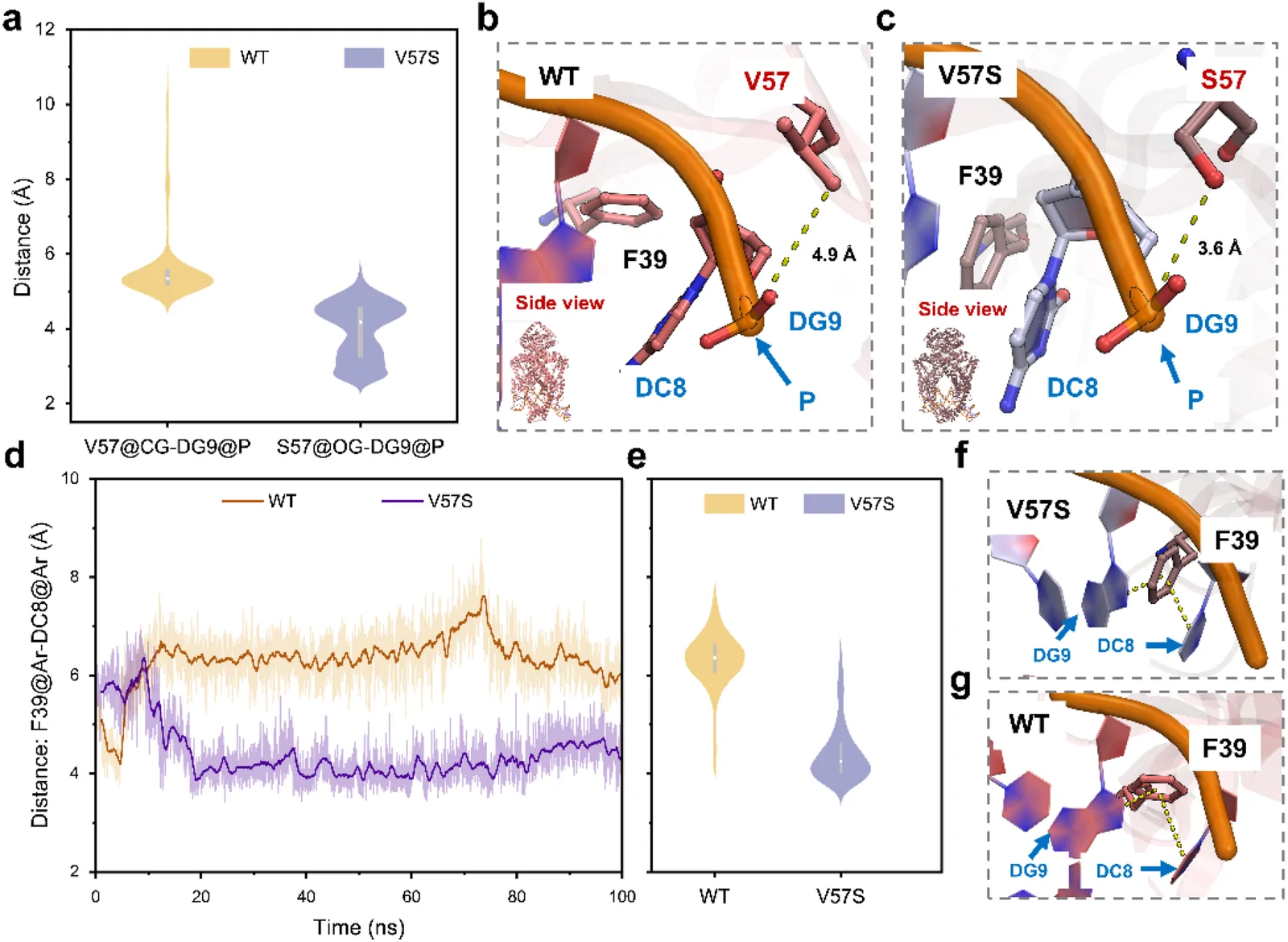
Interaction analysis of V/S57 and F39 with A/G mismatched DNA in WT and V57S systems. The distance distribution between V/S57 and mismatched base DG9 during 100-ns GaMD simulation (a), and the visualized structure of WT (b) and V57S (c). The changes in distance (d) and the distance distribution (e) between the benzene ring of F39 and the base ring of DC8 during 100-ns GaMD simulation, and the visualized structural of V57S (f) and WT (g).

In addition, the D472N mutant lost the ability to distinguish between matched and mismatched DNA and exhibited a high level of non-specific binding (>98%), indicating that this site is particularly critical for the specific recognition of *Taq*MutS. In the WT-matched DNA system, the distance between D472 and the matched DNA was more than 8 Å (Fig. 6a and b). However, the distance between N472 and DNA was less than 4 Å in the D472N mutant (Fig. 6a and c). Moreover, the contact frequency of D472 with the matched DNA was less than 20% in the WT system, while the contact frequency of N472 with the matched DNA exceeded 75% in the D472N mutant (Fig. 6d). This phenomenon was attributed to the formation of new hydrogen bonds between N472 and the phosphate backbone of the matched DNA, which pulled the DNA chain closer to the protein and further disrupted the parallel stacking of DNA double helix. Importantly, the DNA chain in the D472N system tended to move away from F39, resulting in a significant decrease in the contact frequency between F39 and DNA (Fig. 6e and f). Thus, these results demonstrated that D472 plays a crucial role in repelling the DNA chain and maintaining the specific recognition of DNA by *Taq*MutS. The negative charge of D472 may repel the negatively charged phosphate backbone of DNA, thereby pushing the DNA chain closer to F39 (a key residue for mismatched DNA recognition) and facilitating the specific binding of *Taq*MutS to the mismatched DNA. To verify whether this effect was dependent on the negatively charged side chain, the D472E mutant was constructed. As shown in Fig. 3k, D472E restored the capacity to recognize the mismatched DNA, confirming that the DNA-repelling effect mediated by the negatively charged side chain is essential for the mismatched DNA recognition of *Taq*MutS. Collectively, these results demonstrated that the distance between the residue 472 and mismatched DNA was inversely correlated with the distance between the residue 57 and mismatched DNA (Fig. 7a–c). Specifically, the mismatched DNA was positioned closer to S57 and F39 but farther from D472 in the V57S mutant, which exhibited enhanced mismatched DNA discrimination (Fig. 7b). The mismatched DNA was located farther from V57 and F39 but closer to N472 in the mutant D472N, which failed to distinguish between the perfectly matched and mismatched DNA (Fig. 7c). Thus, we proposed that S57 and D472 synergistically regulate the distance between F39 and mismatched DNA through a push-pull mechanism, thereby modulating the mismatched DNA recognition of *Taq*MutS.

**Fig. 6:**
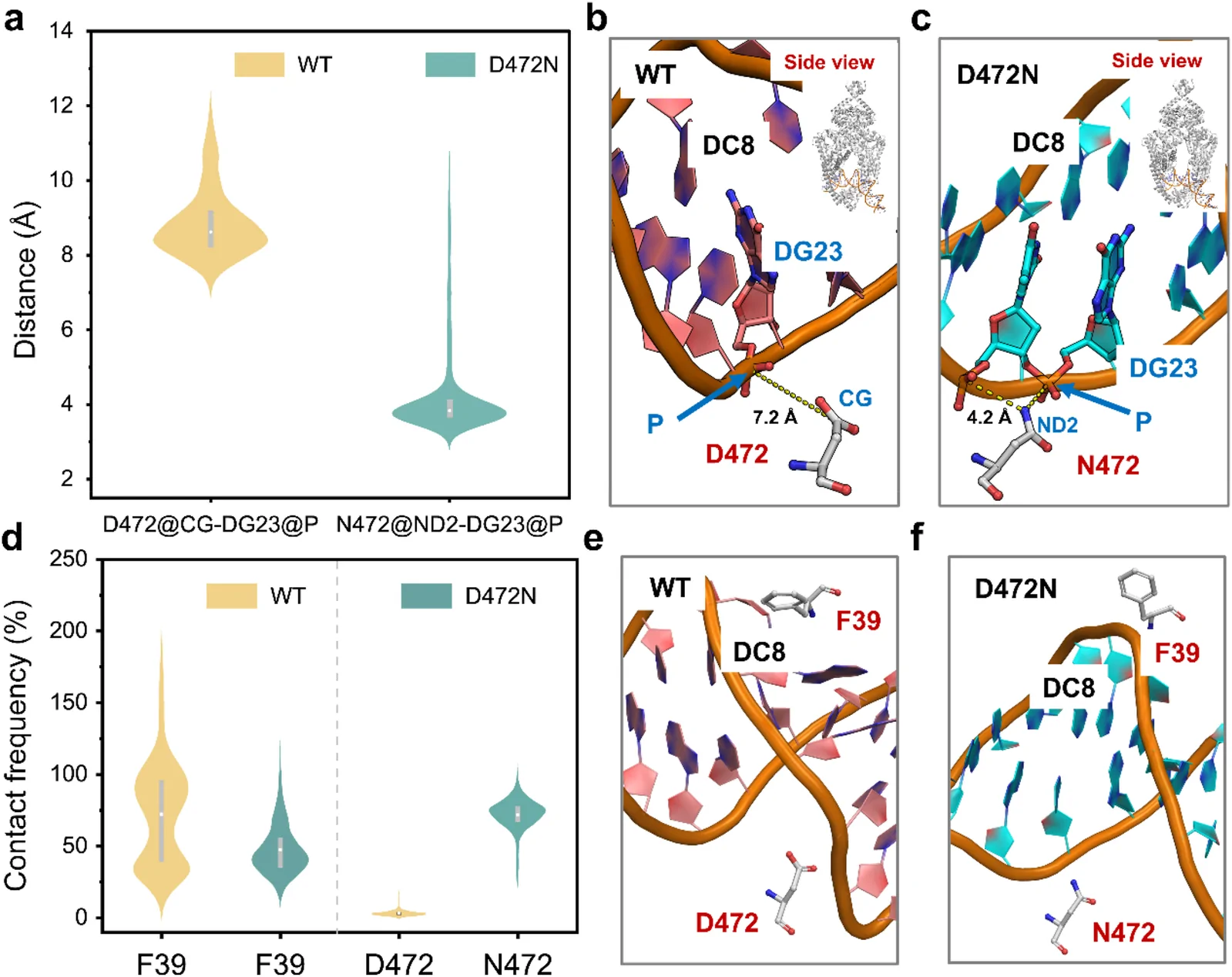
Interaction analysis of D/N472 and F39 with correctly matched DNA. The distance distribution between D/N472 and base DG23 during 100-ns GaMD simulation (a), and the visualized structure of WT (b) and D472N (c). The contact frequencies of F39 and D/N472 with the correctly paired DNA during 100-ns GaMD simulation (d) and the visualized structure of WT (e) and D472N (f).

**Fig. 7:**
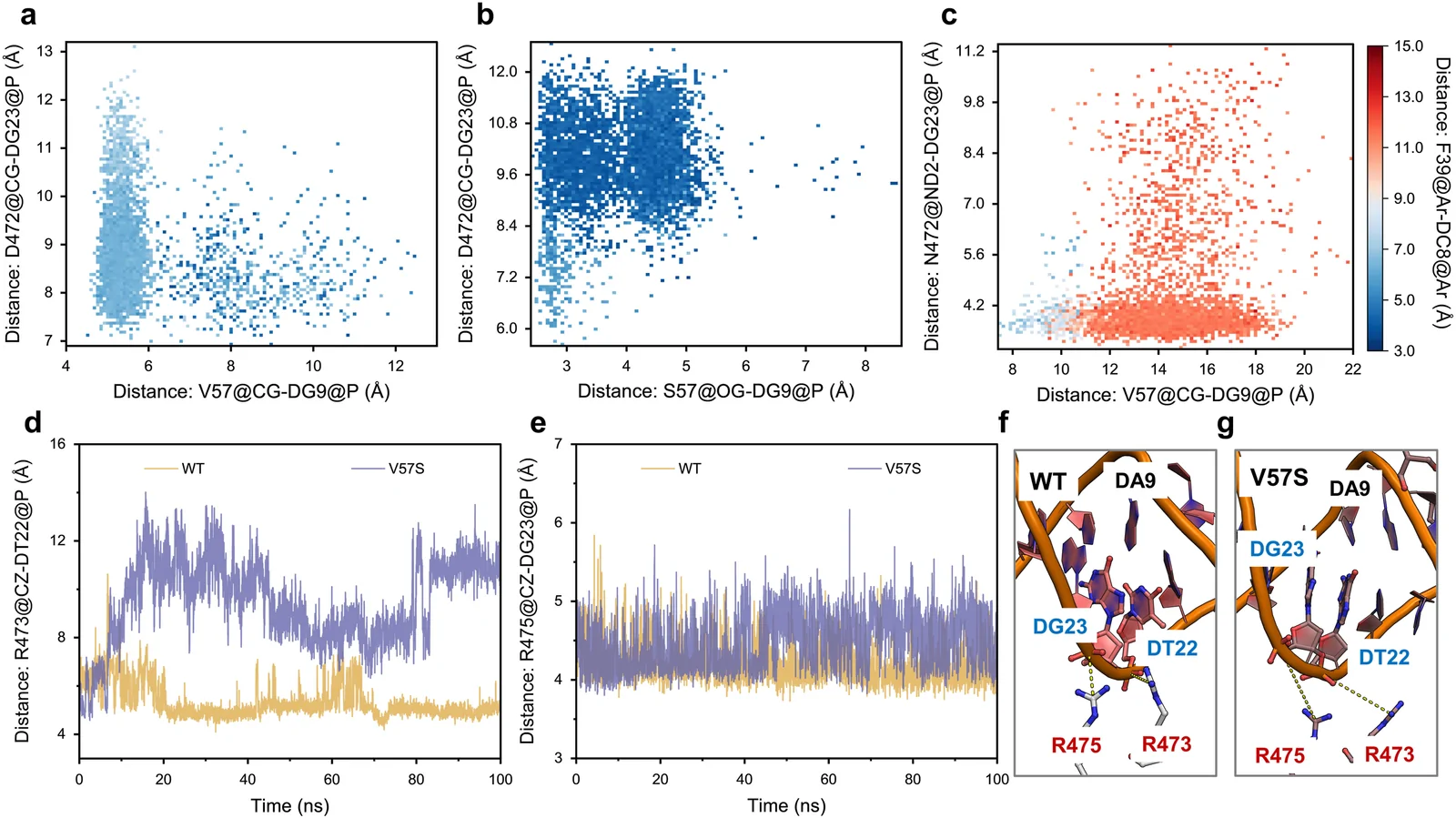
Analysis of the synergistic interaction of S57 and D472, and the mechanism of nonspecific binding of V57S. The distance distribution between V/S57 and DG9, D/N472 and DG23, as well as F39 and DC8 in the system of WT (a), V57S (b), and D472N (c) with A/G mismatched DNA during 100 ns GaMD simulations. The change in distance between R473 (d) and R475 (e) and the base loops of DT22 and DG23, respectively, in the system of WT and V57S with correctly paired DNA, along with the visualized structures of WT (f) and V57S (g).

### 3.6 Regulation of non-specific recognition of TaqMutS by electrostatic synergistic effect

To explore the molecular mechanism underlying the reduced non-specific binding of V57S, the complex systems were constructed for 100-ns GaMD simulations, in which WT and V57S bind to the correct DNA, respectively. The results showed that R473 and R475 formed stable hydrogen bonds with DNA in the WT-matched DNA complex (Fig. 7d–f). Specifically, the distance between R473@CZ and DT22@P ranged from 4 to 5 Å, and the distance between R475@CZ and DG23@P was maintained at approximately 4 Å, indicating stable hydrogen bond interactions between these arginine residues and the matched DNA. In contrast, the distances between R473@CZ and DT22@P as well as between R475@CZ and DG23@P, were 7–12 Å and 5 Å in the V57S-matched DNA complex, respectively (Fig. 7d–g). These results indicated that the hydrogen bond interactions between arginine and the matched DNA were either lost or significantly weakened, leading to a reduced binding ability of V57S to the matched DNA. Additionally, the binding rate of K61R mutant to the matched DNA increased from 39.6% to 71.8% (Fig. 3h), demonstrating that the K61 site was involved in regulating the non-specific binding of *Taq*MutS. Contact frequency analysis revealed that K61 had a lower contact frequency with the DNA base moiety but formed stable hydrogen bonds with the DNA phosphate backbone to stabilize DNA structure in the WT complex (Fig. 8a–c). In contrast, R61 exhibited a higher contact frequency with the DNA bases but no interaction with the phosphate backbone in the K61R complex. This phenomenon might be attributed to the longer side chain of arginine, which caused steric hindrance and thus prevented the formation of hydrogen bonds with the DNA phosphate backbone (Fig. 8a–d). Since K/R61 and R473 were located on the opposite sides of DNA (Fig. S10), they exhibited competitive binding to the DNA phosphate backbone. In the WT complex, the distance between R473@CZ and DT22@P was more than 5 Å, whereas this distance was reduced to approximately 4 Å in the K61R complex (Fig. 8e–g). These results indicated that due to the lack of electrostatic interactions with R61, the matched DNA instead formed hydrogen bonds with R473 on the opposite side, thereby enhancing the non-specific binding of *Taq*MutS. To verify this mechanism, arginine was mutated to histidine to generate the mutant K61H, which has a positively charged side chain with smaller steric hindrance. In the K61H complex, the distance between R473@CZ and DT22@P ranged from 5 to 8 Å (Fig. 8e and h), indicating that the hydrogen bond between R473 and the matched DNA was eliminated. Correspondingly, the binding rate of K61H to the matched DNA decreased to below 20% (Fig. 3l). In summary, these results confirmed that K61 was involved in the non-specific binding of *Taq*MutS by regulating the electrostatic interactions with DNA. Specifically, R473 could form stable hydrogen bonds with DNA, thereby promoting the non-specific binding of *Taq*MutS; K61 competitively bound to the DNA phosphate backbone through its positively charged side chain, keeping DNA away from R473 and reducing the non-specific binding.

**Fig. 8:**
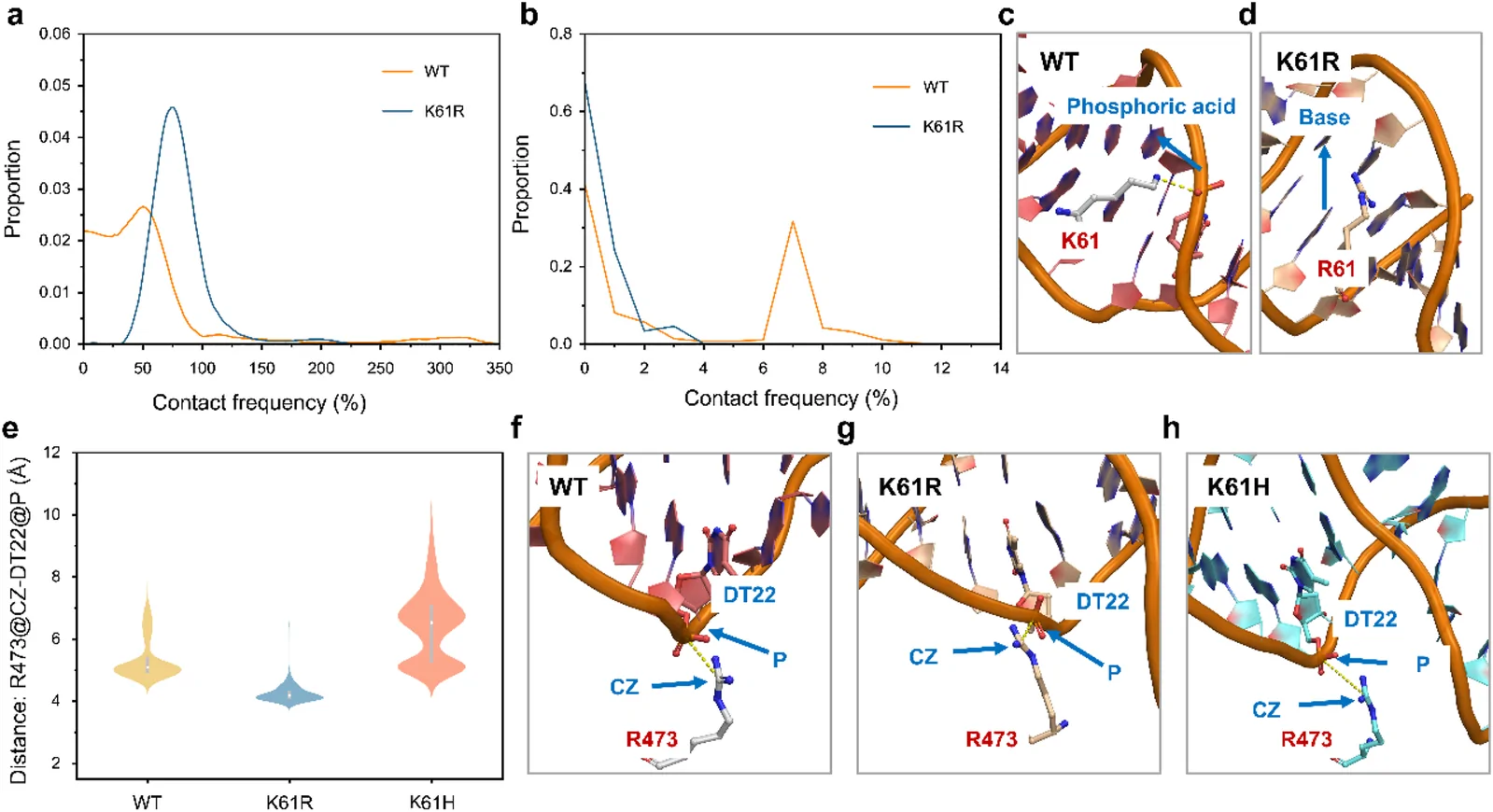
Analysis of the mechanism of electrostatic synergy between K61 and R473. The frequency distributions of K/R61 contacts with the base portion (a) and the phosphate group portion (b) of DNA, respectively, in the system of WT and K61R with correctly paired DNA, and visualized structural of WT (c) and K61R (d). The distance distribution between R473 and DT22 during 100 ns GaMD simulation (e), and visualized structural of WT (f), K61R (g), and K61H (h).

### 3.7 Mismatched DNA binding and error-removal performance of V57S and K61H

To evaluate whether the introduced mutations affected the mismatched DNA binding performance of *Taq*MutS, we characterized the mismatched DNA binding behaviors of V57S and K61H under varying temperatures and prolonged storage conditions. In the range of 20–70 °C, all the proteins maintained comparable mismatched DNA binding efficiencies. The mutants V57S and K61H exhibited binding efficiencies exceeding 80%, moderately higher than WT (Fig. S11). Following the storage at 4 °C for 24 days, both variants retained nearly unaltered mismatched DNA binding capacities (Fig. S12), indicating their favorable storage stability. Collectively, these results revealed that the two single-point mutations did not compromise the intrinsic mismatched DNA binding performance or storage stability of *Taq*MutS.

To verify the practical performance of V57S and K61H, a defined DNA pool of perfectly-matched and mismatch-bearing sequences was subjected to the *Taq*MutS-mediated error-removal assay (Fig. 9a). Prior to the error-removal assay, the amount of magnetic beads was optimized for efficient capture of *Taq*MutS. As shown in Fig. 9b, the concentration of unbound proteins decreased with the step-wise increasing bead volume. A bead volume of 40 μL was sufficient to achieve complete protein capture and was therefore selected for the subsequent error-removal assay. Following the error-removal assay, the DNA collected from the supernatant was analyzed by next-generation DNA sequencing to quantify the fractions of perfectly-matched DNA and four distinct mismatched DNA substrates (Fig. 9c). In the untreated DNA pool, mismatched DNA sequences accounted for 18.2% of total sequencing reads. The four mismatched substrates exhibited initial fractions of 3.5% (A/G), 3.9% (G/T), 3.8% (Deletion), and 7.0% (T/C). Following the treatment with WT, the fractions of four mismatched types declined to 2.5% (A/G), 2.5% (G/T), 1.1% (Deletion), and 2.4% (T/C). Notably, marked reductions in all the mismatched categories could be detected for the V57S variant, with fractions of 1.4% (A/G), 1.9% (G/T), 1.7% (Deletion) and 1.7% (T/C). Comparable trends were observed for K61H, where the abundance of each mismatched type decreased to 1.6% (A/G), 1.7% (G/T), 1.3% (Deletion), and 1.7% (T/C). Compared with WT, both V57S and K61H exhibited better performance in recognizing and removing most types of mismatched DNA, except for the Deletion substrate. These findings suggested that the mutants V57S and K61H are promising error-correction tools for DNA synthesis and DNA information storage, where the synthesis-derived sequence errors frequently impair the sequence fidelity and limit the scalability of DNA data libraries. These variants could alleviate the error accumulation and improve the overall reliability of synthetic DNA substrates for such scenarios.

**Fig. 9:**
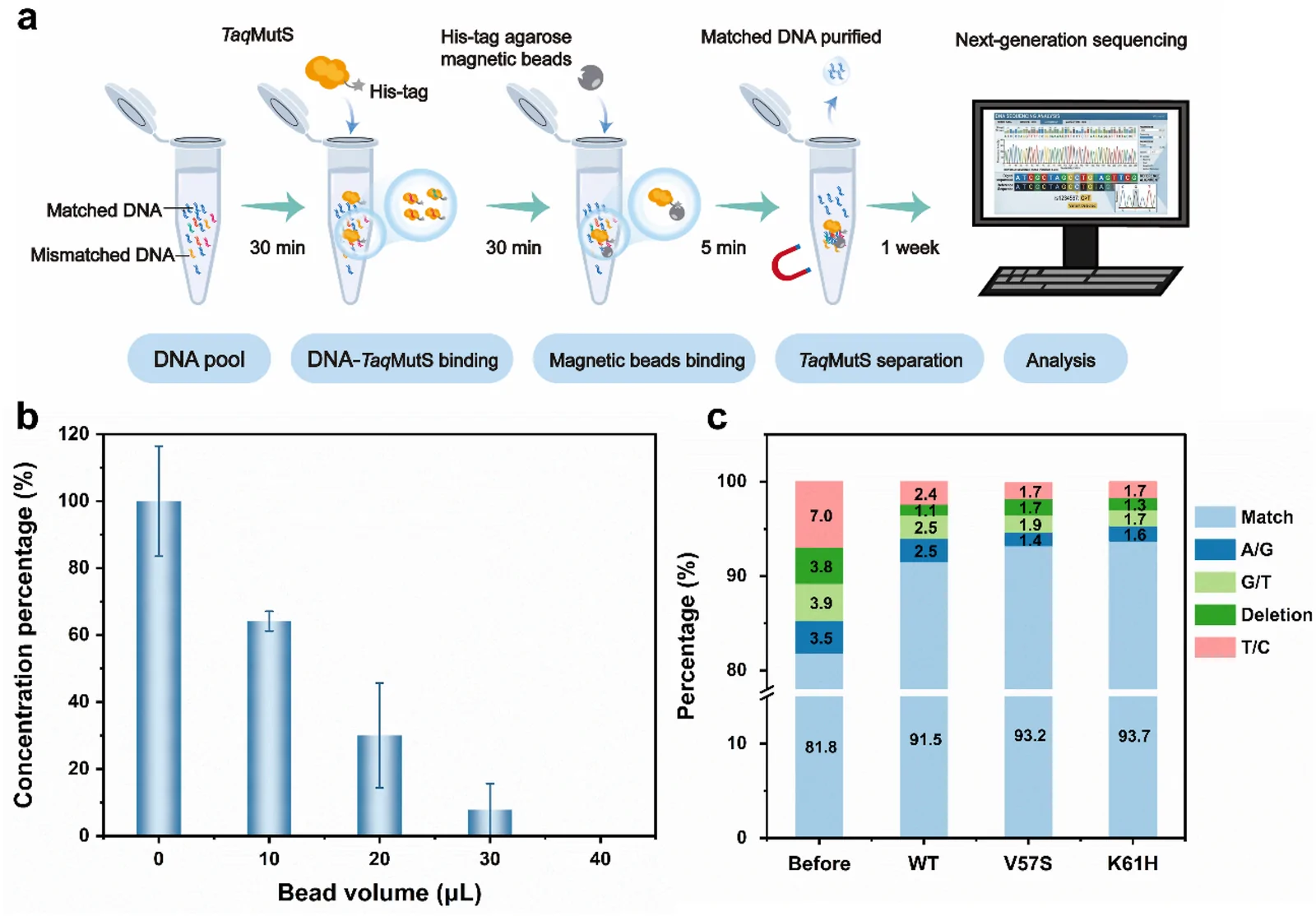
Error-removal performance assessment. (a) Schematic diagram of the *Taq*MutS-mediated error-removal assay. (b) Optimization of magnetic bead amount for the *Taq*MutS capture. Data are presented as mean values ± standard deviation of triplicate experiments. (c) Sequencing-based quantitative profiling of the defined DNA pool before and after the treatment with WT, V57S, or K61H.

## 4 Conclusion

In conclusion, alanine scanning mutagenesis, homologous substitution mutation, and GaMD simulation were systematically integrated to decipher the detailed molecular mechanism of mismatched DNA recognition by *Taq*MutS. We identified that the local conformational strain of duplex DNA acts as the fundamental structural determinant for specific mismatched DNA discrimination of *Taq*MutS. Specifically, S57 and D472 cooperatively controlled the accessibility of key recognition residue F39 *via* a push-pull mechanism: S57 formed new hydrogen bonds with DNA to stabilize the π-stacking interaction between F39 and the mismatched bases, while D472 regulated the DNA orientation through electrostatic repulsion to facilitate the mismatched DNA detection. Meanwhile, K61 and R473 synergistically suppressed the non-specific binding of *Taq*MutS to perfectly matched DNA through electrostatic effects. Accordingly, V57S and K61H exhibited enhanced recognition specificity. Their binding efficiencies to A/G mismatched DNA reached 98.4% and 92.7%, while the binding affinities to perfectly matched DNA declined to 28.2% and 16.9%, respectively. Further, the mutants V57S and K61H displayed improved mismatched DNA removal capacity in the customized DNA-pool error-removal assay. Overall, this work elucidated the intrinsic mismatched DNA recognition mechanism of *Taq*MutS, providing theoretical guidance for the rational design of high-specificity mismatched DNA recognition proteins to overcome the issues of DNA synthesis errors in DNA information storage.

## CRediT authorship contribution statement

**Wenyan Jiang:** Writing – original draft, Methodology, Investigation, Formal analysis, Data curation, Conceptualization. **Yiyang Ding:** Writing – original draft, Visualization, Software, Investigation. **Haojie Sun:** Resources, Methodology, Investigation, Data curation. **Yuxuan Li:** Validation, Software, Formal analysis. **Xiao Liang:** Writing – review & editing, Supervision, Project administration, Funding acquisition. **Quanshun Li:** Writing – review & editing, Supervision, Resources, Funding acquisition.

## Declaration of competing interest

The authors declare that they have no known competing financial interests or personal relationships that could have appeared to influence the work reported in this paper.
