# Supplementary material for: Computational and experimental analysis for the mismatched DNA recognition mechanism of TaqMutS
Source: Synth Syst Biotechnol. 2026 Nov 4;19:124–34. doi: 10.1016/j.synbio.2026.10.011 (PMC13756796; doi:10.1016/j.synbio.2026.10.011)
Supplement: Supplementary file 1 [file mmc1.docx]

**Supplementary Information**

Computational and experimental analysis for the mismatched DNA recognition mechanism of *Taq*MutS

**Figure S1.** SDS-PAGE analysis of *Eco*MutS expressed in *E. coli* BL21(DE3). Lane M: protein marker; lane 1: uninduced bacterial cell; lane 2: IPTG-induced bacterial cell; lane 3: sediment of IPTG-induced bacterial cell lysate; lane 4: supernatant of IPTG-induced bacterial cell lysate; lane 5: effluent fraction of the supernatant obtained from the lysate of IPTG-induced bacteria; lane 6: the fraction eluted with 10 mM imidazole; lane 7: the fraction eluted with 50 mM imidazole; lane 8: the fraction eluted with 150 mM imidazole; lane 9: the fraction eluted with 500 mM imidazole.

**Figure S2.** SDS-PAGE analysis of *Taq*MutS expressed in *E. coli* BL21(DE3). Lane M: protein marker; lane 1: uninduced bacterial cell; lane 2: IPTG-induced bacterial cell; lane 3: sediment of IPTG-induced bacterial cell lysate; lane 4: supernatant of IPTG-induced bacterial cell lysate; lane 5: effluent fraction of the supernatant obtained from the lysate of IPTG-induced bacteria; lane 6: the fraction eluted with 50 mM imidazole; lane 7: the fraction eluted with 150 mM imidazole; lane 8: the fraction eluted with 500 mM imidazole.

**Figure S3**. SDS‑PAGE analysis (a) and protein concentration quantification (b) of purified *Taq*MutS mutants.

**Figure S4**. Structural and aggregation‑propensity analyses of WT and D472A. Local structural environment surrounding the residue 472 in WT (a) and D472A (b). The hydrogen bond between D472 and R473 in WT is marked by a yellow dashed line, with the corresponding distance labeled. Insets show the viewing orientation of each structural representation. (c) Predicted residue-level solubility profiles of WT and D472A spanning the residues 468-476. (d) Comparison of TA of aggregation profile and Na4vSS between WT and D472A.

**Figure S5.** Non-denaturing polyacrylamide electrophoresis analysis of *Taq*MutS mutants for their binding ability to different mismatched DNA: (a) V36A; (b) V57A; (c) K61A; (d) R76A; (e) R473A; (f) V36M; (g) V57S; (h) K61R; (i) R76H; (j) D472N; (k) D472E; (l) K61H.

**Figure S6.** Hydrogen bonds between DC8, DA9, DC10, and their paired bases during 100-ns GaMD simulation of WT and the correctly paired DNA system.

**Figure S7.** Probability distribution of π-stacking angles between WT and the DC8, DA9, and DC10 base loops within the correctly paired DNA system during 100-ns GaMD simulation.

**Figure S8.** The changes in distance and the distance distribution between the F39 benzene ring and the DG9 base ring within the WT, V57S and mismatched DNA systems during 100-ns GaMD simulation.

**Figure S9.** The changes in distance and the distance distribution between the F39 benzene ring and the DA9 base ring within the WT and correctly paired DNA system during 100-ns GaMD simulation.

**Figure S10.** Schematic representation of the spatial distribution of R61 and R473 in the mutant K61R.

**Figure S11**. Temperature-dependent mismatched DNA binding of WT, V57S and K61H. Binding rates were determined using A/G mismatch‑bearing DNA as the substrate. (a, c, e) Representative gels for mismatched DNA binding assays. (b, d, f) Quantified binding rates for each protein at different temperatures.

**Figure S12**. Storage stability assessment of WT, V57S and K61H. Binding rates were determined using A/G mismatch‑bearing DNA as the substrate. (a) Representative gels in mismatched DNA binding assays. (b) Quantified binding rates for each protein at different storage time.

**Table S1.** Primer sequences for the construction of mutants.

| Mutants | Primers |
| --- | --- |
| V36A | CTTGTTGTTCCAGGCCGGTGATTTCTACGAG CTCGTAGAAATCACCCGCCTGGAACAACAAG |
| V36M | CTATCTCTTGTTGTTCCAGATGGGTGATTTCTACGAGTGC GCACTCGTAGAAATCACCCATCTGGAACAACAAGAGATAG |
| V57A | GCGCTAGGACTGGCGCTGACCCATAAAAC GTTTTATGGGTCAGCGCCAGTCCTAGCGC |
| V57S | GTGCGCTAGGACTGAGCCTGACCCATAAAAC GTTTTATGGGTCAGGCTCAGTCCTAGCGCAC |
| K61A | CTGGTCCTGACCCATGCGACCAGCAAAGACTTC  GAAGTCTTTGCTGGTCGCATGGGTCAGGACCAG |
| K61R | GACTGGTCCTGACCCATCGTACCAGCAAAGACTTCAC GTGAAGTCTTTGCTGGTACGATGGGTCAGGACCAGTC |
| K61H | GACTGGTCCTGACCCATCATACCAGCAAAGACTTCAC GTGAAGTCTTTGCTGGTATGATGGGTCAGGACCAGTC |
| R76A | CGGGTATCCCGTTGGCGGCGTTTGAGGCGTAC GTACGCCTCAAACGCCGCCAACGGGATACCCG |
| R76H | GGTATCCCGTTGCATGCGTTTGAGGC GCCTCAAACGCATGCAACGGGATACC |
| D472A | GTTCAGACCTTGAAAGCGCGTCAGCGTTACACC  GGTGTAACGCTGACGCGCTTTCAAGGTCTGAAC |
| D472N | CCAGTTCAGACCTTGAAAAATCGTCAGCGTTACACCCTTC GAAGGGTGTAACGCTGACGATTTTTCAAGGTCTGAACTGG |
| D472E | CAGACCTTGAAAGAACGTCAGCGTTACACC GGTGTAACGCTGACGTTCTTTCAAGGTCTG |
| R473A | CAGACCTTGAAAGACGCGCAGCGTTACACCCTTC GAAGGGTGTAACGCTGCGCGTCTTTCAAGGTCTG |

**Table S2.** The sequence of mismatched DNA.

| DNA | Sequences |
| --- | --- |
| Match-F | TATCCAGCTGCCAGTCACCAGTGTCAGCGT |
| Match-R | ACGCTGACACTGGTGACTGGCAGCTGGATA |
| C/T-R | ACGCTGACACTGGTGATTGGCAGCTGGATA |
| G/T-R | ACGCTGACACTGGTGACTTGCAGCTGGATA |
| G/A-R | ACGCTGACACTGGTGACTAGCAGCTGGATA |
| A/G-R | ACGCTGACGCTGGTGACTGGCAGCTGGATA |
| T/C-R | ACGCTGACACTGGTGACCGGCAGCTGGATA |
| Deletion-R | ACGCTGACACTGGTGA_TGGCAGCTGGATA |

**Table S3**. The sequence of mismatched DNA for error-removal assay.

| DNA | Sequences |
| --- | --- |
| Match-F-59 | GCGGACTATTTAACATATCCAGCTGCCAGTCACCAGTGTCAGCGTATCGGCCTTGCTCC |
| Match-R-59 | GGAGCAAGGCCGATACGCTGACACTGGTGACTGGCAGCTGGATATGTTAAATAGTCCGC |
| A/G-R-59 | GGAGCAAGGCCGATACGCTGACGCTGGTGACTGGCAGCTGGATATGTTAAATAGTCCGC |
| G/T-R-59 | GGAGCAAGGCCGATACGCTGACACTGGTGACTTGCAGCTGGATATGTTAAATAGTCCGC |
| T/C-R-59 | GGAGCAAGGCCGATACGCTGACACTGGTGACCGGCAGCTGGATATGTTAAATAGTCCGC |
| Deletion-R-59 | GGAGCAAGGCCGATACGCTGACACTGGTGA_TGGCAGCTGGATATGTTAAATAGTCCGC |
